# A comparative evaluation of quantum machine learning architectures for breast cancer classification using clinical and genomic data

**DOI:** 10.3389/frai.2026.1837513

**Published:** 2026-07-29

**Authors:** Saartak Allena, Smrithy G. S., Balaji Chandrasekaran

**Affiliations:** 1 School of Computer Science and Engineering, Vellore Institute of Technology, Chennai, India; 2 Department of Computer Science and Engineering, College of Engineering, Guindy, Anna University, Chennai, India

**Keywords:** breast cancer, classical baselines, cross-validation, medical benchmarking, METABRIC, quantum computing, quantum machine learning

## Abstract

**Introduction:**

In recent years, high-dimensional clinical and genomic data have gained significant importance for prognosis and personalized medicine in breast cancer. But the use of quantum machine learning (QML) on such data is limited by the availability of few qubits, the computation time of quantum simulation, and dimensionality reduction. This work systematically compares several QML architectures for breast cancer classification in the presence of realistic and simulator constraints.

**Methods:**

The experiments were performed on a dataset of METABRIC breast cancer patients (2,509 patients). After handling missing values and one-hot encoding, there were 63 features in the processed data set. The feature space was reduced by Principal Component Analysis (PCA) to 12, 4 and 2 components for the implementations of quantum computers, respectively, with 56.10 ± 0.14%, 26.78 ± 0.57% and 16.23 ± 0.33% of the variance retained. Three QML models were tested: Quantum Neural Networks (QNN), Quantum K-Nearest Neighbors (QKNN), and Quantum Support Vector Machines (QSVM), with the models being simulated. Seven classical classification models were tested: Logistic Regression, SVM with RBF kernel, K-Nearest Neighbors, Random Forest, XGBoost, LightGBM and Multilayer Perceptron, both with PCA-matched and full 63-feature representation. All primary results are reported with 5-fold cross validation.

**Results:**

Among the evaluated QML architectures, QKNN using 12 principal components achieved the strongest performance, attaining an accuracy of **75.11% ± 3.76%**, an F1-score of **0.7088 ± 0.0433**, and a ROC-AUC of **0.8148 ± 0.0394**. Even though all of the QML models performed significantly poorly in comparison to classical models trained on the entire 63-feature data set, the latter models were able to achieve about **94% accuracy** with XGBoost, Random Forest, and Logistic Regression. The comparison also showed the effect of information loss due to PCA is significant in predictive performance in both classical and quantum models.

**Discussion:**

The results show that for high dimensional breast cancer data, currently available simulator-based QML models can learn meaningful patterns with limited quantum resources, but are not as effective as powerful classical machine learning models when complete feature representations are available. The study does not report any sort of quantum advantage or clinical use, but rather a benchmark of current QML architectures that has been rigorously calculated and repeated, with a focus on the impact of dimensionality reduction, validation approaches, and simulator limitations, as well as outlining challenges that need to be overcome prior to practical implementation on real quantum hardware.

## 1 Introduction

Breast cancer has been the cause of death to still about a million women worldwide and is classified as the leading cause of death caused by cancer. Meanwhile, it is estimated that there are approximately 2.3 million new cases of breast cancer every year. The detection role is that it should be timely, accurate and plays a vital role in enhancing patient outcomes and survival rates. Techniques of diagnosis in the past are highly challenged as per and pertaining to the management of the complicated clinical and molecular data that accompanies the modern healthcare setting. Machine learning is no longer merely used in medical diagnosis but it has turned into a groundbreaking technique, where even the analyses with extremely high dimensionality that involve both the genomic and the clinical data can be automatically performed. The classical methods of machine learning were very potent; however, in most cases, they demand significantly large quantities of computational capacities and storage facilities when they are applied to the genomic data which define the state-of-the-art cancer research.

The METABRIC dataset which contains clinical and genomic data of 2,509 breast cancer patients collected through the Molecular Taxonomy of Breast Cancer International Consortium is well suited for the testing of novel classification methods. The classical machine learning techniques for breast cancer prediction have been discussed in-depth through the lens of the METABRIC dataset and similar sources. The recent hybrid methodologies that combine transfer learning with traditional classifiers have pushed the performance limits, albeit often at the cost of elevated model complexity and extra compute requirements.

There are promising attempts to make sense of learning problems in the context of a computational paradigm that may be able to provide useful representations of some learning problems, including through superposition, entanglement, and quantum feature maps. However, much of the current quantum machine learning research in healthcare remains on the exploratory side, particularly when users test the models on classical simulators instead of real quantum machines. With medical datasets, there is a need for robust conclusions to be reached with careful comparisons to contemporary classical baselines, explicit exploration of dimensionality reduction, and testing protocols for assessing stability that are based on various data partitions, and so on. So, for this reason, this study does not make such large and unsubstantiated claims on quantum speed up or clinical superiority. It rather seeks an empirical controlled evaluation of QML architectures given the limitations of the current simulator as well as qubit capacity.

Quantum Neural Networks, which use parameterized quantum circuits for variational learning, have been looked at for classification problems in medical fields and have been especially good at dealing with very high-dimensional data by means of quantum feature maps. Quantum Kernel Methods, including Quantum Support Vector Machines, use quantum feature spaces to identify intricate decision boundaries that might be too hard for classical kernels and recent studies have shown that they perform well on medical datasets comparable to those used for classical methods. Quantum K Nearest Neighbors uses quantum fidelity as a distance metric and this might give its output better quality than that from classical methods in some of the feature spaces through the use of quantum state overlap calculations. Quantum Generative Adversarial Networks extend adversarial learning into quantum circuits. Nevertheless, these advancements are quite encouraging; on the other hand, still, there is a lack of extensive comparisons between several quantum architectures and robust classical baselines, particularly when it comes to the issue of trade offs between information compression, model size, and classification performance.

This study makes four contributions. First it introduces and tests several QML architectures: QNN, QKNN and QSVM architectures, on the METABRIC dataset while maintaining explicit qubit limits and PCA restrictions. Second, it takes the classical comparison one step further than simply one Random Forest, but utilises seven classical baselines: Logistic Regression, SVM-RBF, KNN, Random Forest, XGBoost, LightGBM and MLP. Third, it looks at the effect of information loss due to PCA by testing two classical models: the first with PCAs matched, the second with the full 63 features. The comparison is somewhat fairer, since the classical models are tested under two different conditions. Fourth, it replaces the previous single train-test division with stratified 5-fold cross-validation, and it also provides probabilistic metrics calibration metrics as well as statistical agreement measures and simulator wall-clock timing. For the QGAN discriminator, it is reserved to explore limitations as a research exercise only – since the adversarial training budget was too limited to produce consistent behavior, it is not used as a primary support to the comparative conclusions.

Five primary QML setups are evaluated: QNN with 12 qubits and either 2 layers or 4 layers, QKNN with 4 qubits or 12 qubits, and QSVM with 2 qubits. The QGAN discriminator is not included as this is an exploratory negative result. The classical side is also evaluated with seven baselines in the PCA-matched and full-feature settings. The paper discusses and evaluates accuracy, precision, recall, F1-score, specificity, balanced accuracy, AUC-ROC, AUC-PR, log loss, Brier score, Cohen’s Kappa, Matthews Correlation Coefficient, Negative Predictive Value, Diagnostic Odds Ratio, Youden’s Index, simulator inference time, and model storage requirements.

## 2 Materials and methods

### 2.1 Dataset description and preprocessing

The study utilizes the METABRIC (Molecular Taxonomy of Breast Cancer International Consortium) dataset, comprising clinical and genomic profiles of 2,509 breast cancer patients. The classification task is formulated as a binary outcome predicting patient survival status.

The entire data set had been 39 clinical and molecular attributes. Those features that were missing more than 10% of the data, such as Tumor Stage and 3-Gene Classifier Subtype were excluded. All values missing were replaced with median values for continuous variables and mode imputation for categorical variables. The first category was dropped to prevent multicollinearity, and the 63 categorical features were transformed into a 63-dimensional feature space using one-hot encoding.

The validation protocol uses stratified 5-fold cross-validation instead of just a single 80:20 split. For each fold, the preprocessing routine, like imputation, encoding, scaling and PCA, is trained only on the training portion, and then that same pipeline is pushed onto the validation partition to avoid data leakage. Also a fixed random seed 42 was chosen to help reproducibility, and keep results consistent across reruns.

### 2.2 Dimensionality reduction using PCA

Due to exponential scaling constraints in quantum simulation, Principal Component Analysis (PCA) was applied to reduce feature dimensionality. PCA transforms the original feature space into orthogonal components ranked by variance.

Three configurations were used:

12 components (12 qubits, 56.10 ± 0.14% variance retained across folds).
4 components (4 qubits, 26.78 ± 0.57% variance retained across folds).
2 components (2 qubits, 16.23 ± 0.33% variance retained across folds).

Each PCA model was fitted only on the training partition of each cross-validation fold and subsequently applied to the corresponding validation partition. The number of kept components directly sets the qubit count that gets used by each QML model. This setup enables a fair comparison between QML models and PCA-matched classical models while also allowing full-feature classical models to quantify the impact of information loss introduced by PCA.

### 2.3 Quantum data encoding

Classical feature vectors were encoded into quantum states using angle encoding. Each PCA component was mapped to a rotation parameter of quantum gates (RY rotations), enabling efficient state preparation with low circuit depth.

This encoding strategy was selected due to its compatibility with Noisy Intermediate-Scale Quantum (NISQ) devices and its ability to represent high-dimensional data with minimal circuit complexity.

### 2.4 Quantum machine learning models

#### 2.4.1 Quantum neural network (QNN)

Two QNN architectures were implemented using 12 qubits with different circuit depths (2-layer and 4-layer configurations). Each model consists of angle embedding followed by parameterized rotation layers and CNOT-based entanglement.

The 4-layer QNN has 48 trainable rotation parameters, while the 2 layer version ends up with 24 trainable rotation parameters. In the implementation, the QNN outputs were turned into trainable logits, and then optimized using Adam, with binary cross entropy loss over about 30 epochs. This setup gives probabilistic outputs, which is why we can report AUC-ROC AUC-PR log loss and Brier score, across 5 fold cross validation.

#### 2.4.2 Quantum K-nearest neighbors (QKNN)

QKNN computes distances using quantum fidelity:


$$d(x_{i},x_{j})=1-|\psi (x_{i})|{\psi (x_{j})|}^{2}$$

Two configurations were evaluated:

12 qubits (12 PCA components).

4 qubits (4 PCA components).

Predictions were obtained using the five nearest neighbors (k = 5). In the experiments, the QKNN outputs were converted, from deterministic majority voting to distance weighted class probabilities based on the quantum fidelity distance. This approach enables consistent computation of probabilistic metrics, including ROC-AUC, PR-AUC, log loss, and Brier score.

#### 2.4.3 Quantum support vector machine (QSVM)

QSVM employs a quantum kernel defined as:


$$K(x_{i},x_{j})=|\phi (x_{i})|{\phi (x_{j})|}^{2}$$

Owing to computational constraints, a two-qubit feature map was employed. The kernel matrices were then computed, and fed into a classical SVM setup, specifically with a precomputed quantum fidelity kernel. Probability estimation was turned on too, to help with probabilistic metrics and for the calibration analysis.

#### 2.4.4 Quantum generative adversarial network (QGAN)

A QGAN framework was implemented with parameterized quantum circuits for both generator and discriminator. The discriminator was used as a classifier after adversarial training.

The training ran with Adam optimization for 5 epochs, simply because we hit computational limits. However, the limited adversarial training budget resulted in unstable discriminator behavior and poor convergence. So, QGAN is no longer used as part of the main comparative evidence, instead it stays only as an exploratory limitation.

### 2.5 Classical baseline model

The study uses this expanded classical baseline suite, rather than just Random Forest only. Logistic Regression, SVM with an RBF kernel, K Nearest Neighbors, Random Forest, XGBoost, LightGBM, and a multilayer perceptron were looked at. The experiment consisted of two cases: Case A, where the classical models were trained with the same PCA reduced features on which the QML models were trained, and Case B, where the classical models were trained with the 63 features without PCA. That sort of structure explicitly separates the information that PCA might drop or compress from how the models behave.

### 2.6 Experimental setup

All experiments were brought together in Python, using PennyLane for the quantum circuits and Scikit-learn compatible tooling for the classical models. All quantum models were evaluated using classical simulation or analytical quantum-fidelity kernels.

For the main models we employed a relatively standard preprocessing procedure using a fixed seed and stratified 5-fold cross validation. The results are presented as mean ± standard deviation of the folds. The evaluation of all models was performed in an identical preprocessing pipeline, feature representation and validation procedure to ensure fair comparison.

### 2.7 Evaluation metrics

Seventeen evaluation metrics were used across four categories:

Classification metrics: Accuracy, Precision, Recall, F1 score, Specificity, Balanced Accuracy.
Probabilistic metrics: AUC-ROC, AUC-PR, Log Loss, Brier Score.
Statistical metrics: Cohen’s Kappa, MCC, NPV, DOR, Youden’s Index.
Computational metrics: Inference Time, Model Size.

This comprehensive framework enables multi-dimensional evaluation of predictive performance, reliability, calibration, and efficiency.

### 2.8 Validation strategy

Stratified 5 fold cross validation was used to evaluate the model performance. Stratified 5-fold cross-validation was adopted to obtain more reliable performance estimates than a single train-test split.

To minimize any leakage of the data, the imputation, encoding, scaling and PCA steps were performed on the training partition within each fold only. Thereafter, mean ± standard deviation was used to summarize data for the wise results, and Wilcoxon signed rank tests were utilized for the pairwise comparisons of QML.

## 3 Related work

Machine-learning-based breast cancer diagnosis and prognosis has become a widespread topic of research in the last ten years, especially given that the use of large-scale clinical and genomic datasets like METABRIC is not new. Machine learning models such as Support Vector Machines, Random Forests and deep neural networks that are based on classical models have shown to be strong predictors using handcrafted clinical features and high-dimensional genomic data. Recent publications have investigated ensemble learning, deep learning networks, to enhance survival prediction and subtype classification, typically yielding high accuracy at the price of increased model complexity, large memory footprint, and high computational demands particularly with tasks on genomic feature space of higher dimensions (Mahmoud et al., 2024; Mufidah et al., 2025; Bavisetti et al., 2025; Kishore Khan et al., 2024).

As quantum computing has emerged as a potential alternative paradigm, Quantum Machine Learning (QML) has been suggested as a possible alternative paradigm that can handle high-dimensional data with the use of quantum state representations. Initial research in quantum healthcare systems focused largely on Quantum Support Vector Machines and Quantum Neural Networks on small-scale or image data, and showed proof-of-concept viability but not clinical functionality (Eutamene et al., 2025; Ovi et al., 2025). Medical imaging and signal processing tasks have also been explored regarding quantum Convolutional Neural Networks and hybrid quantum and classical architectures, with possibilities of expressibility and nonlinear feature generation being realised by constrained feature representations (Ovi et al., 2025; Hanafi and Ali, 2025).

Medical imaging has also explored hybrid quantum classical adversarial systems, which have shown to be possible with limited quantum resources (Hanafi and Ali, 2025). Later quantum distance- and kernel-based approaches, including Quantum K-Nearest Neighbors and quantum-enhanced kernel learning, have demonstrated comparable performance on smaller medical datasets and the role of quantum fidelity in reflecting the behavior of complex feature interactions (Eutamene et al., 2025). Nevertheless, most of these experiments consider only a single quantum architecture and idealized simulations and most commonly provide measurements based on accuracy. Exhaustive tests incorporating probabilistic calibration, statistical tolerance, information loss to dimensionality reduction and computational efficiency have not found much presence in the literature.

Moreover, there are little studies that explicitly examine trade-offs between the number of qubits, circuit depth, information storage, and model accuracy on clinical data on real-world clinical data. Unlike other works, the current study offers a comprehensive and systematic analysis of various quantum machine learning systems, such as QNN, QKNN, QSVM and QGAN on METABRIC breast cancer data. This study fills important gaps in the existing understanding of quantum healthcare studies by using a rich portfolio of seventeen evaluation metrics, explicitly quantifying the effect of the information bottlenecks caused by PCA, and the circuit depth and quantum resource limits. The article is not a claim that quantum machine learning is the instantaneous substitution of classical algorithms, but a promising new addition that its advantages and drawbacks should be studied and comprehended under extremely realistic circumstances.

## 4 Methodology

### 4.1 Dataset description and preprocessing

We adopted a stratified 5-fold cross validation technique rather than an 80:20 train–test split. This helps to keep a similar class distribution for each fold. To prevent data leakage, all pre-processing methods such as scaling and dimensionality reduction were done only on the training part of each fold and then applied to the part of the fold that was used for validation. This configuration is more reliable and will allow less variability that may occur with one split.

Preparing the data involved a multistep preprocessing protocol tailored to quantum machine learning applications. The first phase of quality control pinpointed the features that were highly missing, in that, their null values constituted over 10 percent. Two features “3 Gene classifier subtype” and “Tumor Stage” were the ones to be excluded since they both were having this problem. The continuous variables in the features that were still present and had missing values were filled with the median while the categorical ones were mode imputed. Categorical features underwent one-hot-encoding transformation with the first category dropped to eliminate the multicollinearity artifacts. After applying these transformations, the feature space was processed to the dimensionality of 63.

All experiments were conducted using stratified 5-fold cross-validation. Stratified sampling secured class distributions to be the same for both partitions. All models were implemented with a random seed of 42 to ensure that the experiments could be repeated. The last preprocessing step performed StandardScaler normalization on all 63 features separately. Most importantly, scaling factors used in test data were taken from training only, which avoided data leakage and kept experiment validity intact.

### 4.2 Principal component analysis for dimensionality reduction

The 63 dimensional feature space is a huge computational problem for the current quantum hardware simulators, thus, in that case, the use of Principal Component Analysis is necessary to get the dimension reduced. PCA is a key preprocessing step for quantum implementations, as it not only reduces the complexity of quantum circuits but also keeps the original data’s informative content at the highest level. This requirement emerges because of the memory required for simulating the quantum circuits which grows exponentially with the number of qubits.

For our PCA implementation, we used the scikit-learn library but made different transformations depending on the quantum architecture. The three PCA configurations that were set up are 12 components for 12 qubits, 4 components for 4 qubits, and 2 components for 2 qubits. Each transformation was trained only on the training set, and so the test set was transformed using the parameters learned from the training set only. This method ensures that the test set does not provide information about the training set, which could affect the evaluation of the test set and compromise the validity of the generalization judgment.

The mathematics behind it all involves calculating the covariance matrix, and then decomposing it into its eigenvalues and eigenvectors, which gives the principal components sorted by the amount of variance they explain. The PCA conversion to the k dimension for a data point x that exists in R^63 is:

z = WT(x – *μ*) with the matrix W being the top k principal components (63 × k matrix) and μ being the mean vector of the training set (63 × 1 matrix).

In the variance retention analysis, we examine how much information is preserved under different PCA configurations. Across the cross-validation folds, the 12-component transformation retained 56.10 ± 0.14% of the original variance, while the 4-component and 2-component setups preserved 26.78 ± 0.57% and 16.23 ± 0.33%, respectively. These results clearly indicate that PCA introduces a substantial information bottleneck. Therefore, it should be considered a key experimental limitation rather than a routine preprocessing step.

One of the components has a contribution that shows a monotonic decrease in the variance capture across the spectrum. The first principal component is responsible for about 12.5 percent of the overall variance and this is in fact the most significant data pattern. The second component gives around 8.3 percent and the following ones do it more and more in a diminished manner. This development corroborates that the first few components extract the predominant variance, while the remaining ones are just able to identify the more finely-distributed information.

The experiments explicitly evaluate the effect of this PCA bottleneck by comparing PCA-matched and full-feature classical baselines. Results indicate that the full feature classical models manage roughly 94 percent accuracy, but the PCA 12 classical models only get around 82 percent accuracy. Meanwhile the top QML model reaches just 75.11 percent accuracy. So this confirms that trimming the dimensionality is a big cause behind the performance downturn.

PCA use, however, was driven by several practical considerations. Quantum circuit simulation has an exponential memory complexity scaling of O(2^n), which means that to simulate a 63 qubit system, one would need 2^63 complex amplitude values, making it impossibly expensive for the current computing power. Today’s quantum simulators have practical operating limits set around 12 to 20 qubits. Moreover, with higher qubit counts comes the greater risk of noise, and deeper circuits that are needed for high dimensional feature processing will suffer even more from error rates. From the optimization point of view, PCA helps in speeding up training convergence by allowing to work on a smaller variety of parameters, i.e., the low variance components that might contain noise rather than actual signal are eliminated.

In the context of quantum machine learning, each retained principal component directly maps to a single qubit through angle encoding. Consequently, the number of PCA components determines the number of qubits required for quantum state preparation. This one-to-one mapping will impose a hard limit on the dimensionality of the features kept, thus not only providing a dimensionality reduction but an architectural constraint that is imposed due to hardware limitations and simulators in use today.

### 4.3 Quantum neural network (QNN) with 12 qubits

To explore the correlation between circuit depth and model expressiveness, two QNN architectural variants were realized. The two versions are built on the same fundamental architecture, but differ solely in the number of layers.

The QNN 12Q 4 L architecture utilizes a quantum circuit of 12 qubits along with 4 layers of parametrized rotations and entanglements. The circuit is constructed starting from an AngleEmbedding encoding layer that maps the 12 PCA components to the rotation angles of qubits, and then it is followed by 4 variational layers. The first layer performs single-qubit RY rotations on all qubits, followed by a CNOT entanglement connection that sequentially links neighboring qubits. The full parameter space has 48 trainable weights, which are calculated as 4 layers multiplied by 12 qubits. The measurement is done using the Pauli Z operator to get the expectation value from qubit 0, which results in outputs that fall within the range [−1, 1]. Angle-based quantum feature encoding is adopted due to its low circuit complexity and suitability for NISQ-era implementations, as supported by recent comparative studies on quantum encoding strategies for biomedical data (Eutamene et al., 2025).

The QNN 12Q 4 L model got trained with the Adam optimizer and a binary cross-entropy loss, with probabilistic logits in the mix. Training ran for 30 epochs, with a learning rate scheduler. The updated implementation shows fold by fold validation results and probabilistic metrics, not just one lone split. Because quantum circuit simulation is computationally intensive, training was performed sequentially under controlled computational setting.

The mathematical representation of the quantum circuit for each layer is as follows:


$$U(x,\theta )=(\prod_{l=1}^{L}U_{\mathrm{ent}}U_{\mathrm{rot}}(\theta_{l}))U_{\mathrm{enc}}(x)$$

Where, U_enc_(x) indicating the angle embedding operation, U_rot_(θl) standing for the parameterized rotation layer with weights θl, and Uent being the entanglement layer consisting of CNOT gates.

The QNN 12Q 2 L version is built on the same architectural concept but has less depth as it uses only 2 layers instead of 4. Hence, there are 24 parameters to be trained (calculated as 2 layers × 12 qubits). The other hyperparameters remain the same as for the 4-layer implementation. This shallower design will help to understand whether the lower depth of the circuit is a factor in the training difficulty caused by barren plateau phenomena and whether the expressibility remains at adequate levels.

### 4.4 Quantum K nearest neighbors (QKNN)

QKNN utilizes quantum fidelity as its distance metric, which might give it the upper hand over the classical approach in the case of certain feature geometries. The computation of QKNN-style distance based on quantum fidelity between data points x_i and x_j can be written as:


$$\mathrm{dQ}(x_{i},x_{j})=1-|\psi (x_{i})|{\psi (x_{j})|}^{2}$$

Where, ∣*ψ*(x)⟩ is the quantum state encoding of the feature vector x. Overall the fidelity measures the probability of quantum states’ overlap, thus 0 for the states being orthogonal and 1 for them being identical, in turn mapping the distance from 0 (for identical samples) to 1 (for maximally divergent samples).

The QKNN 12Q setup uses twelve qubits corresponding to twelve PCA components, and the neighbor parameter was set to k = 5. In the revised evaluation, the predictions rely on distance weighted neighbor probabilities not merely majority voting, so metrics like AUC-ROC AUC-PR, log loss and Brier score get computed.

During the prediction stage of the QKNN method, the quantum fidelity distance between the validation observation and all the training observations in the fold is computed. The algorithm identifies the k nearest training samples and estimates class probabilities using inverse-distance weighted voting. This update addresses the previous situation where it was hard to clearly identify any probabilistic measure of QKNN, which is why the probabilistic scores are meaningful in this circumstance.

The QKNN 4Q variant sticks to the same methodology but works with 4 qubits and processes the 4 component PCA reduced feature space. This dimensionality reduction not only speeds up the distance calculations but also results in an information loss where only 26.64 percent of the original variance is kept.

### 4.5 Quantum support vector machine (QSVM)

QSVM is a quantum kernel method that allows to discover the intricate decision boundaries. The similarity between data points in a quantum feature space is quantified by the quantum kernel:


$$K(x_{i},x_{j})=|\phi (x_{i})|{\phi (x_{j})|}^{2}$$

Where, ∣*ϕ*(x)⟩ represents the quantum feature map produced by the quantum circuit.

The QSVM 2Q model employs two qubits to encode the two-component PCA representation. For that, the quantum feature map applies angle encoding and from this, a precomputed quantum fidelity kernel comes out. After that, the kernel is handed to a classical SVM classifier. Also, probability estimation is switched on, so that AUC-ROC, AUC-PR, log loss and Brier score can be computed.

During training, the kernel matrix K_train is built from all training samples within each fold. Then Scikit-learn’s SVC, with a precomputed kernel, solves the dual optimization task. Even though the kernel is quantum-inspired, the runtime that gets reported mostly mirrors classical kernel computation plus the usual SVM overhead, not actual physical quantum hardware execution, you know.

During the hunt for predictions, the kernels between the test and training samples are computed producing K_test ∈ R^(n_test × n_train). The final predictions are then made by:


y(x)=sign(∑iαiyiK(x,xi)+b)

Kernel matrix calculation is limited to 2 qubits thereby managing computational expense which increases quadratically with the training set size growth.

### 4.6 Quantum generative adversarial network (QGAN)

Quantum Generative Adversarial Networks have recently been explored for learning complex medical data distributions using quantum circuits, motivating the inclusion of a QGAN discriminator in this study (Solanki et al., 2024). QGAN employs a tactic of adversarial training wherein the quantum generator is always in the opposite camp with respect to the quantum discriminator network, and the latter is trained first and then used for classification. The challenging circuit has for its part the AngleEmbedding for the introduction of the real data, followed by parameterized RY rotations on all qubits of the circuit (12) and CNOT tortuosity entanglement patterns. The measurement is done by extracting the expected value of Pauli Z on the 11th qubit, which results in a probabilistic output distinguishing between real and fake classifications.

The circuit for the generator takes the opposite path, starting from the introduction of Hadamard gates that create superposition states in all qubits, followed first by parameterized RY rotations and then CNOT entanglement. The generator’s measurement gives through all qubits the 12-dimensional output vectors.

The adversarial training protocol is conducted as follows. The first step is the input data preprocessing done by MinMaxScaler which ensures that all features are within the interval [−1,1] that is necessary for the proper quantum state preparation. During each training epoch, the discriminator’s task is to distinguish the real data from the one generated; it achieves this by driving down the cost function:


LD=−log(D(xreal))−log(1−D(G(z)))

The next step is the training of the generator to trick the discriminator, whereby the generator’s cost function LG = −log(D(G(z))) is minimized. Both networks are optimized using the Adam optimizer at a learning rate of 0.05.

The QGAN experiment was treated more as an exploratory test rather than a core result. With only five epochs of adversarial training, the setup did not provide enough room for stable convergence. In fact, during the original run, the discriminator fell into a degenerate classification mode — essentially getting stuck in a strange behavior pattern. Because of these limitations, the QGAN outcomes were not used to support any claims about the competitiveness of quantum machine learning in the paper.

### 4.7 Classical baselines and validation protocol

To address the limitations in classical benchmarking, the experiments include a range of models: Logistic Regression, SVM with RBF kernel, KNN, Random Forest, XGBoost, LightGBM, and MLP. These are tested in two settings — one using PCA-reduced features and another using the full set of 63 features. The PCA-matched setup provides a fairer comparison with the quantum models since they operate in a reduced feature space. Meanwhile, the full-feature evaluation highlights the trade-off: it shows the performance penalty introduced by dimensionality reduction directly.

### 4.8 Evaluation metrics

The assessment of models made use of a thorough metric portfolio that consisted of 17 measures divided into five categories. This multidimensional assessment framework provides information much more than the accuracy measurement, including capturing of statistical reliability, probabilistic calibration and computational efficiency characteristics required for medical diagnosis applications.

Six key measures were used to develop the main classification metrics. Accuracy is the overall degree of rightness, it is the sum of the true positives and true negatives divided by the sum of the true positives and true negatives plus the false positives and false negatives, or the percentage of samples that are correctly classified across both classes. Precision, which is also referred to as positive predictive value (PPV), is computed as TP/(TP + FP), and it assesses the percentage of positive forecasts that are correct. Recall also known as sensitivity or true positive rate (TPR) is calculated as TP/(TP + FN), and it is used to represent the % of true positives that are detected. Specificity is similar to TNR, with TN/(TN + FP) indicating the degree of specificity of the model. The one that gives the harmonic mean of precision and recall is F1 score and it is calculated as 2(Precision × Recall)/(Precision + Recall), which is a fair indicator if there is an imbalance between the two classes. Balanced accuracy is an arithmetic average of sensitivity and specificity and it is used as a performance evaluator that is especially suitable for imbalanced medical datasets, providing class balanced evaluation.

In a probabilistic metric context, four types of metrics, were applied to assess the reliability and discrimination power of the prediction. AUC ROC is the area under the Receiver Operating Characteristic curve, indicating the ability of the model to rank the positive samples above the negative ones over all classification thresholds. The closer the values to 1.0 the better the discrimination; however, 0.5 is the case of random guessing performance. AUC PR is the area under Precision-Recall curve and is useful for imbalanced data sets when there is a significantly lower number of positive class instances. Log Loss is expressed as follows: mathematically:


−1/N(∑(yilog(pi)+(1−yi)log(1−pi))

is stricter with confident mistakes than with uncertain errors and offers calibration assessment. Brier Score, the formula being 1/N(∑(pi−yi)^2), shows the average squared difference between _the_ probabilities given by the model and the actual ones, and it is the case of lower scores representing better calibration.

The former statistical agreement metrics were made up of five measures which went beyond the raw accuracy to quantify prediction reliability and consistency. Cohen’s Kappa is the measure that determines the extent of the agreement over the chance expectation, and it does so by taking into consideration the chances of correct predictions being made by random guessing thus allowing for the possibility of that happening. When Kappa values are more than 0.60 it means that there is a considerable amount of agreement, while those rated 0.40 to 0.60 means moderate agreement, 0.20 to 0.40 suggest fair agreement, and below 0.20 point to slight agreement. The Matthews Correlation Coefficient (MCC) gives a well-rounded measurement throughout all the confusion matrix elements, it is computed as


TP×TN−FP×FN/sqrt((TP+FP)(TP+FN)(TN+FP)(TN+FN))

The range of MCC is from negative 1 through 0 (such as random guessing) to positive 1 (full agreement), this property of it has made it particularly suitable for use in the case of imbalanced datasets. The Negative Predictive Value (NPV) is calculated as TN/(TN + FN), it shows how many of the negative predictions are really negative as a percentage. The Diagnostic Odds Ratio (DOR) is calculated as (TP × TN)/(FP × FN), and it gives the ratio of the chances that a test result will be positive in a diseased individual as compared to a non-diseased one. Youden’s Index is expressed as Sensitivity + Specificity − 1 and indicates an area where the model has not caused any misclassifications, with a scale of 0 (no ability to tell the classes apart) to 1 (perfect ability to tell the classes apart).

Two metrics were used for computations to track two practical aspects of deployment. Average inference time: wall clock prediction time per sample, in the simulator or in the analytical kernel. Notably, the times are not used to indicate the actual quantum hardware performance and should not be interpreted as evidence of quantum speedup. While compactness is a measure of the size of the model, it does not necessarily reflect clinical benefit or greater computational power.

The common procedure for all experiments is to set the random seeds, undertake 5 fold Cross Validation, and to preprocess the data at the fold level. All software used were publicly available libraries to ensure that results can be reproduced. Unless stated otherwise, hyperparameters and pre-processing steps were set the same for similar models.

## 5 Results and analysis

### 5.1 Performance metrics across all models

**Figure 1:**
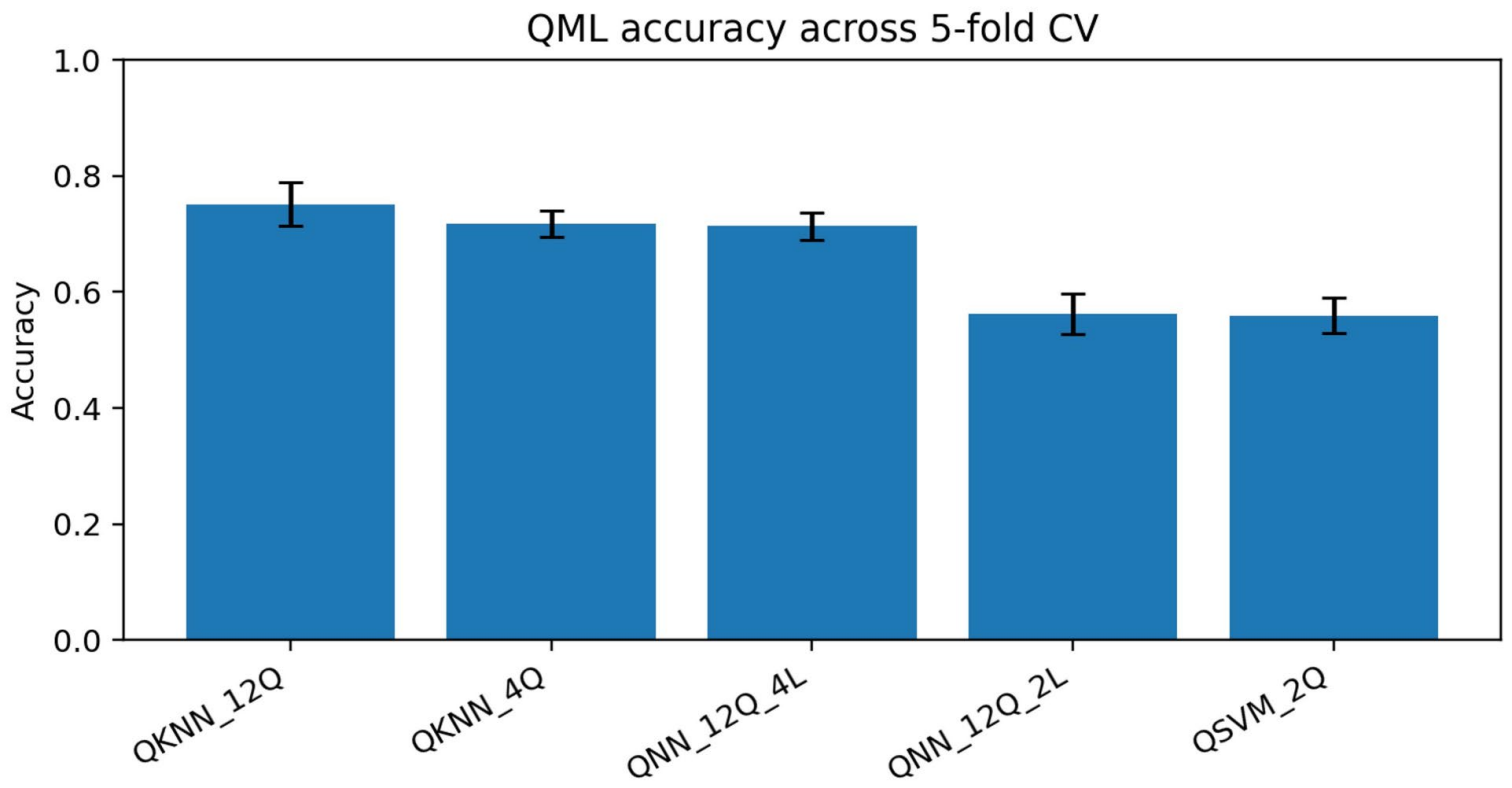
Quantum model comparison based on mean accuracy ± standard deviation across stratified 5-fold cross validation.

Stratified 5 fold cross validation was used to evaluate five major QML configurations and seven classical baseline configurations. The main empirical lesson is that even with PCA and qubit limits, the signal in the predictive models of QML is measurable. When the full feature set is given to classical baselines, however, they beat the simulator based QML models by a considerable margin. The results are therefore best understood as a benchmark rather than demonstrating quantum benefits (see Table 1; Figure 1).

**Table 1:** Classical baseline results under full-feature and PCA-matched settings.

Feature setting	Model	Accuracy	F1	ROC-AUC	Log loss
Full 63 features	Logistic regression	0.9393 ± 0.0136	0.9331 ± 0.0149	0.9764 ± 0.0085	0.1680 ± 0.0384
Full 63 features	SVM RBF	0.9319 ± 0.0191	0.9261 ± 0.0205	0.9682 ± 0.0160	0.1997 ± 0.0516
Full 63 features	KNN	0.8044 ± 0.0367	0.7655 ± 0.0428	0.8720 ± 0.0365	0.8970 ± 0.3995
Full 63 features	Random forest	0.9406 ± 0.0198	0.9342 ± 0.0218	0.9804 ± 0.0124	0.2121 ± 0.0286
Full 63 features	XGBoost	0.9407 ± 0.0052	0.9339 ± 0.0054	0.9795 ± 0.0077	0.1575 ± 0.0327
Full 63 features	LightGBM	0.9366 ± 0.0106	0.9295 ± 0.0108	0.9784 ± 0.0087	0.2709 ± 0.0905
Full 63 features	MLP neural network	0.9244 ± 0.0223	0.9138 ± 0.0257	0.9679 ± 0.0159	0.2192 ± 0.0583
PCA-12	Logistic regression	0.8166 ± 0.0256	0.7949 ± 0.0281	0.8964 ± 0.0251	0.4158 ± 0.0523
PCA-12	SVM RBF	0.8240 ± 0.0294	0.7988 ± 0.0325	0.8961 ± 0.0228	0.4034 ± 0.0392
PCA-12	KNN	0.7768 ± 0.0151	0.7368 ± 0.0207	0.8422 ± 0.0199	1.5625 ± 0.2706
PCA-12	Random forest	0.8098 ± 0.0258	0.7724 ± 0.0283	0.8778 ± 0.0223	0.4376 ± 0.0309
PCA-12	XGBoost	0.8091 ± 0.0186	0.7762 ± 0.0215	0.8865 ± 0.0146	0.4213 ± 0.0296
PCA-12	LightGBM	0.8098 ± 0.0287	0.7798 ± 0.0312	0.8834 ± 0.0203	0.5632 ± 0.0895
PCA-12	MLP neural network	0.8044 ± 0.0286	0.7684 ± 0.0324	0.8783 ± 0.0402	0.4397 ± 0.0670

**Figure 2:**
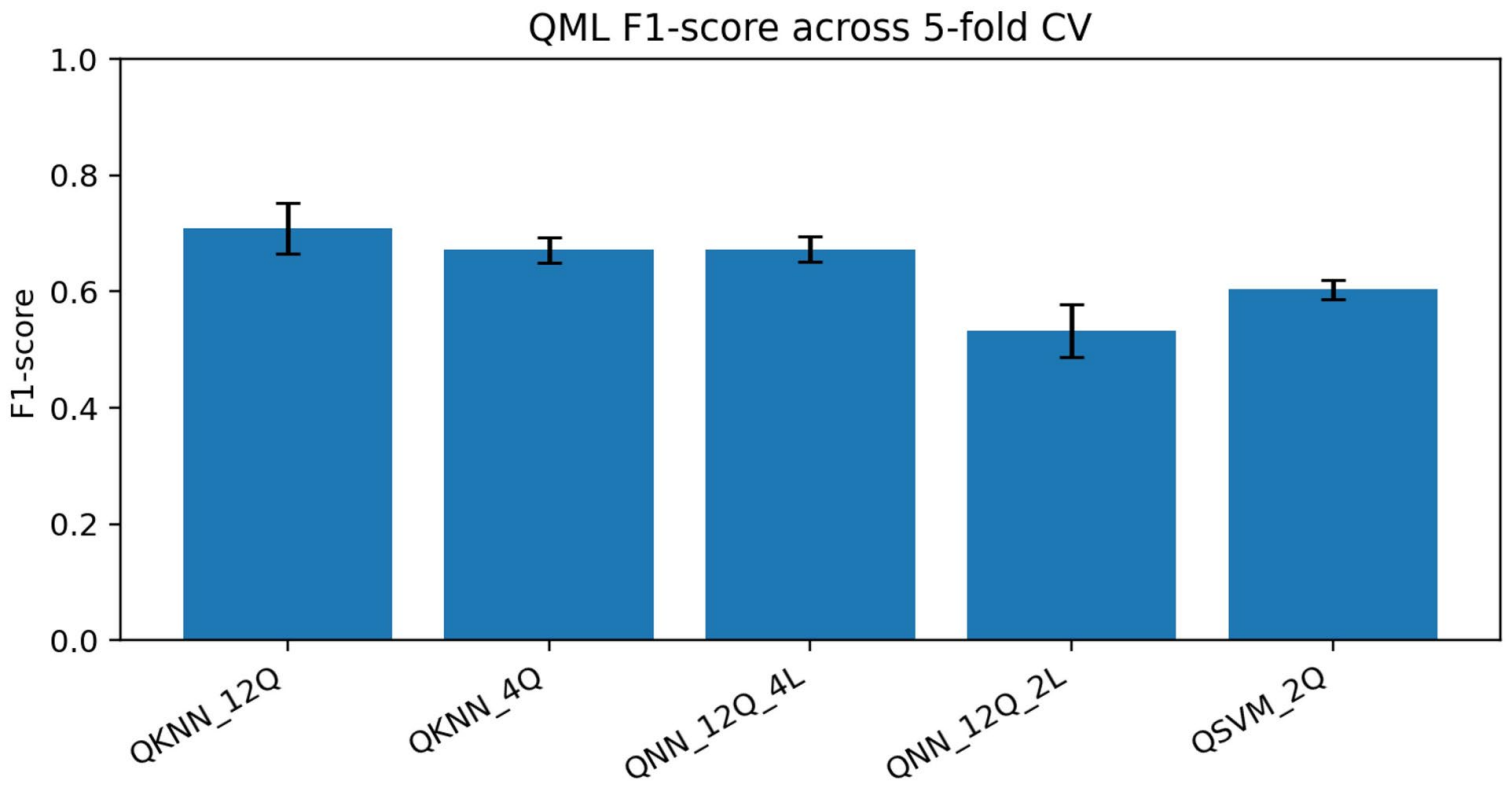
Quantum model comparison based on mean F1-score ± standard deviation across stratified 5-fold cross-validation.

Table 2 shows that QKNN 12Q achieved the strongest QML classification result, with 0.7511 ± 0.0376 accuracy, 0.7088 ± 0.0433 F1 score, and balanced precision recall behavior. QKNN 4Q and QNN 12Q 4 L were lower but still showed meaningful predictive capability. QSVM 2Q and QNN 12Q 2 L were close to weak discrimination, which is consistent with severe PCA compression or limited circuit expressibility (see Figure 2).

**Table 2:** QML core classification metrics under 5-fold cross-validation.

Model	Accuracy	F1	Precision	Recall	Specificity
QKNN_12Q	0.7511 ± 0.0376	0.7088 ± 0.0433	0.7237 ± 0.0497	0.6950 ± 0.0415	0.7945 ± 0.0407
QKNN_4Q	0.7181 ± 0.0224	0.6718 ± 0.0220	0.6836 ± 0.0348	0.6626 ± 0.0388	0.7610 ± 0.0486
QNN_12Q_4L	0.7134 ± 0.0236	0.6730 ± 0.0225	0.6698 ± 0.0305	0.6765 ± 0.0149	0.7419 ± 0.0310
QNN_12Q_2L	0.5623 ± 0.0342	0.5327 ± 0.0462	0.4996 ± 0.0363	0.5776 ± 0.0912	0.5508 ± 0.0866
QSVM_2Q	0.5596 ± 0.0307	0.6034 ± 0.0165	0.4975 ± 0.0240	0.7678 ± 0.0186	0.3991 ± 0.0598

The NaN values in probabilistic metrics do not occur again in the evaluation for QKNN and QSVM. Now, QKNN uses the distance weighted neighbor probabilities, whereas QSVM uses the precomputed kernel SVM to get the probability estimates. In this configuration, metrics like AUC ROC, AUC PR, log loss, and Brier score can be consistently reported across all primary QML models without problems.

Table 3 shows that QKNN 12Q reached the top QML ROC-AUC at 0.8148 ± 0.0394, next is QNN 12Q 4 L at 0.7816 ± 0.0296 and QKNN 4Q at 0.7744 ± 0.0328. Also, QNN 12Q 4 L got the best log loss across the QML set, which hints at better probability calibration than QKNN, even if QKNN kept the edge on accuracy. The QGAN model is included for exploratory purposes and is not considered in the primary comparative conclusions due to limited training stability.

**Table 3:** Probabilistic and calibration metrics under 5-fold cross-validation.

Model	Balanced acc.	AUC ROC	AUC PR	Log loss	Brier score
QKNN_12Q	0.7447 ± 0.0377	0.8148 ± 0.0394	0.7667 ± 0.0566	0.9328 ± 0.1797	0.1766 ± 0.0230
QKNN_4Q	0.7118 ± 0.0205	0.7744 ± 0.0328	0.7037 ± 0.0423	1.1756 ± 0.1239	0.2017 ± 0.0187
QNN_12Q_4L	0.7092 ± 0.0225	0.7816 ± 0.0296	0.7176 ± 0.0375	0.5763 ± 0.0273	0.1944 ± 0.0101
QNN_12Q_2L	0.5642 ± 0.0334	0.5741 ± 0.0446	0.4831 ± 0.0446	0.6890 ± 0.0144	0.2478 ± 0.0070
QSVM_2Q	0.5835 ± 0.0265	0.6099 ± 0.0441	0.5279 ± 0.0593	0.6637 ± 0.0144	0.2360 ± 0.0067

The results displayed in Table 4 indicate QKNN 12Q model has the highest reliability when comparing the QML models, as can be seen from the value of the Kappa index and MCC. Moderate but lesser agreement was shown by QKNN 4Q and QNN 12Q 4 L; and QSVM 2Q and QNN 12Q 2 L remained in the category of slight agreement.

**Table 4:** Agreement metrics and reliability analysis.

Model	Kappa	MCC	NPV	DOR	Youden’s J
QKNN_12Q	0.4917 ± 0.0764	0.4923 ± 0.0767	0.7714 ± 0.0299	9.7904 ± 4.9291	0.4895 ± 0.0753
QKNN_4Q	0.4251 ± 0.0424	0.4263 ± 0.0417	0.7453 ± 0.0180	6.4585 ± 1.2328	0.4236 ± 0.0410
QNN_12Q_4L	0.4179 ± 0.0461	0.4180 ± 0.0460	0.7479 ± 0.0167	6.1671 ± 1.3767	0.4183 ± 0.0450
QNN_12Q_2L	0.1261 ± 0.0659	0.1289 ± 0.0667	0.6298 ± 0.0352	1.7494 ± 0.4208	0.1284 ± 0.0668
QSVM_2Q	0.1575 ± 0.0516	0.1760 ± 0.0515	0.6883 ± 0.0272	2.2338 ± 0.4970	0.1669 ± 0.0530

Pairwise Wilcoxon signed rank test was performed to explore the significance of results at the fold level. It is important to be aware that there were five folds available and so *p* values should be interpreted with caution. The differences for some comparisons were in the direction, but most comparisons were not large enough to extend beyond the commonly accepted 0.05 value. This allows the paper not to claim superiority between QML models.

The chosen statistical tests are summarized to illustrate the significance testing by fold. The overall results do not warrant strong claims in the sense of QML dominance (see Table 5).

**Table 5:** Selected pairwise wilcoxon signed-rank tests for QML models.

Metric	Model 1	Model 2	Wilcoxon statistic	*p*-value
Accuracy	QKNN_12Q	QKNN_4Q	0.0000	0.0625
Accuracy	QKNN_12Q	QNN_12Q_4L	1.0000	0.1250
Accuracy	QKNN_12Q	QSVM_2Q	0.0000	0.0625
F1	QKNN_12Q	QKNN_4Q	0.0000	0.0625
F1	QKNN_12Q	QNN_12Q_4L	2.0000	0.1875
AUC ROC	QKNN_12Q	QNN_12Q_4L	3.0000	0.3125
AUC ROC	QKNN_12Q	QSVM_2Q	0.0000	0.0625

### 5.2 Information loss and performance correlation

PCA information loss has been shown to have an impact on performance and the analysis confirms this. The overall accuracy achieved by the full 63 feature classical model was about 94 percent, while the best PCA 12 classical model was able to achieve 82.40 percent accuracy. Meanwhile the best QML model (QKNN 12Q) remained below around 75.11 percent. So it seems reduced performance of the QML is related to the PCA bottleneck, and not just the quantum model (see Table 6).

**Table 6:** PCA sensitivity based on best classical model per feature setting.

Feature setting	Best classical model	Accuracy	F1	ROC-AUC
Full 63 features	XGBoost	0.9407	0.9339	0.9795
PCA-12	SVM RBF	0.8240	0.7988	0.8961
PCA-4	MLP neural network	0.7633	0.7175	0.8290
PCA-2	KNN	0.6177	0.5373	0.6282

The PCA sensitivity pattern can be seen, if you look at feature settings, which is not surprising. Classical performance seems to drop from around 94 percent on the full 63 feature representation, to 82 percent with PCA-12, then 76 percent with PCA-4, and finally 62 percent when using PCA-2. In other words, the updated manuscript is just not about to imply future full-featured QML accuracy based on PCA-compressed outcomes, that is certainly, not in a easy way. Also, there’s a non-linear relationship between variance retention and performance. The drop between PCA-12 and PCA-4 is not that big, but it’s pretty big between PCA-4 and PCA-2. This essentially demonstrates that the 2 qubit QSVM configuration is primarily a feasibility experiment – not a solid performance baseline.

### 5.3 Impact of quantum circuit depth

When comparing the QNN variants, it is clear that the circuit depth has an effect, although this becomes more pronounced with the modified cross validation protocol. In particular, QNN 12Q 4 L gets 0.7134 ± 0.0236 accuracy and 0.6730 ± 0.0225 F1-score, while QNN 12Q 2 L lands at 0.5623 ± 0.0342 accuracy, and 0.5327 ± 0.0462 F1-score. So yes, it does appear that going deeper helps discrimination, but it does not actually get into the most capable, full feature classical baselines.

The probabilistic metrics essentially convey the same message. QNN 12Q 4 L reaches an ROC-AUC of 0.7816 ± 0.0296, whereas QNN 12Q 2 L is down at 0.5741 ± 0.0446. This suggests that by making the circuit deeper, the rank performance gets better, but only so much, presumably due to compression by PCA and the training pipeline using simulated circuit data. The other side of the coin has a practical component: Deeper circuits may be challenging to train and challenging to simulate. For instance, higher simulator inference time is required by QNN 12Q 4 L than QNN 12Q 2 L, variational circuits are sensitive to the choice of initialization, and optimization can go awry. Overall, the results suggest that the reading is probably a careful trade off, and not a blanket statement that “deeper” circuits are better in all situations.

**Figure 3:**
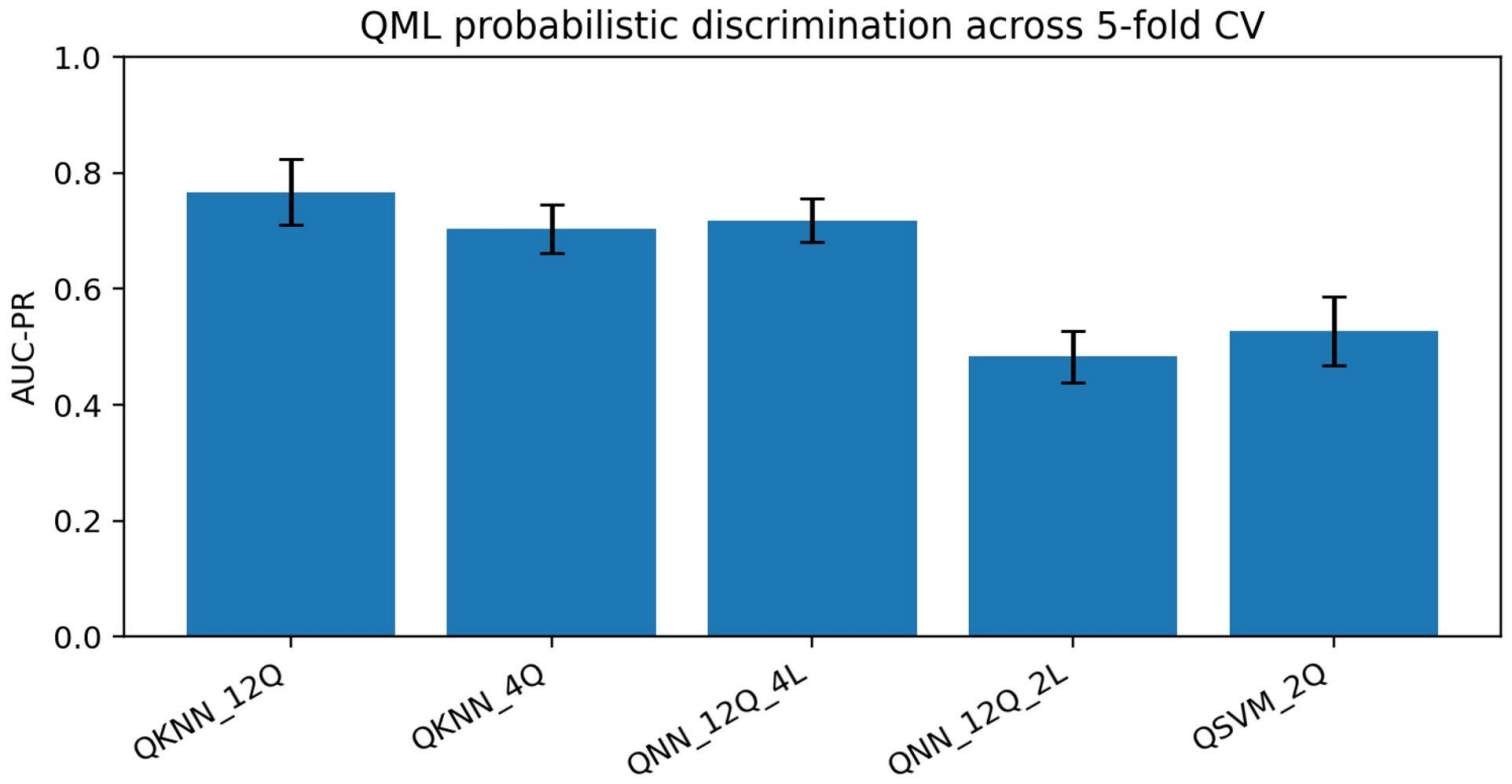
AUC-PR comparison for QML models using probabilistic outputs from the revised 5-fold cross-validation protocol.

Under the hood, the expressibility versus trainability trade-off remains a key problem for QML design. The revised results suggest that using a medium circuit depth can do better than using a shallow one, but this “better” has to be balanced against training cost, simulator latency, and the need to benchmark against strong classical baselines (see Figure 3).

### 5.4 Model size and computational efficiency tradeoffs

The computational metrics in Table 7 must be understood as sort of implementation specific, either simulator wall clock numbers or analytical kernel wall-clock. For example, QNN 12Q 4 L needed around 42.69 ms per validation sample, whereas QNN 12Q 2 L came in at about 23.37 ms. In addition, the QKNN and QSVM timings were lower in the updated analytical kernel implementation, compared with the earlier circuit by circuit simulation. That said, even these values do not mean actual physical quantum hardware speed.

**Table 7:** Simulator timing and model size.

Model	Simulator inference (ms/sample)	Model size (KB)
QKNN_12Q	0.3032 ± 0.0143	120.4938 ± 0.0556
QKNN_4Q	0.1355 ± 0.0087	46.3438 ± 0.0214
QNN_12Q_4L	42.6898 ± 2.2847	0.3906 ± 0.0000
QNN_12Q_2L	23.3727 ± 0.5605	0.2031 ± 0.0000
QSVM_2Q	0.0892 ± 0.0042	39.9820 ± 0.1933

Table 7 sort of indicates that QNN models end up with small parameter storage needs, while QKNN actually keeps the training representation, so the larger model size estimate makes sense. Still, storage compactness is presented as an implementation characteristic not really as proof for deployable clinical superiority, or anything like that.

The main reason behind the timing sensitivity seems to be the simulation environment, plus the expense of computing quantum fidelity distances or kernels. These measurements are kind of handy for comparing the specific pipelines that got implemented, but they should not be taken as evidence that quantum computation is inherently faster or more efficient.

In the interpretation, the inference time values are explicitly labeled as simulator, or analytical kernel wall clock measurements. For anything practical, you’d need real quantum hardware validation, noise modeling, and error mitigation first before you can responsibly state claims about quantum runtime or scalability.

### 5.5 Statistical reliability and prediction consistency

Now, Cohen’s Kappa and MCC are indeed better for conveying reliability than accuracy alone. In this setup, the QKNN 12Q setup(s-of-the-standout!) produced the best metrics out of the QML models with values of 0.4917 ± 0.0764 for Kappa, and 0.4923 ± 0.0767 for MCC. In second and third place came the QKNN 4Q setup and the QNN 12Q 4 L, respectively—they also yield meaningful, albeit somewhat lower, agreement values.

Also, the QNN 12Q 4 L achieved much better results for both metrics than QNN 12Q 2L, indicating that additional layer(s) improve the model’s agreement: not just by a fraction of percentage but rather notably. QSVM 2Q achieved low scores across the board. The major issue in QSVM 2Q comes with its low retention percentage of 16.23 ± 0.33, meaning it operates at extreme compression and hence closer to feasibility analysis rather than learning complex data features. Therefore, this results give additional confidence on the general statement: the models have captured signal under constrained feature spaces, but their agreement metrics alone are insufficient to conclude readiness for clinical applications or to assert dominance over traditional health ML. As for specific behavior by architectural components: QKNN architectures continue to appear to be the dominant flavor of QML on this dataset. Further research direction should perhaps probe for possible uses of quantum fidelity based distance measures, but this is research-area research; it is not an evidence of yet-demonstrated quantum advantage in health care.

**Figure 4:**
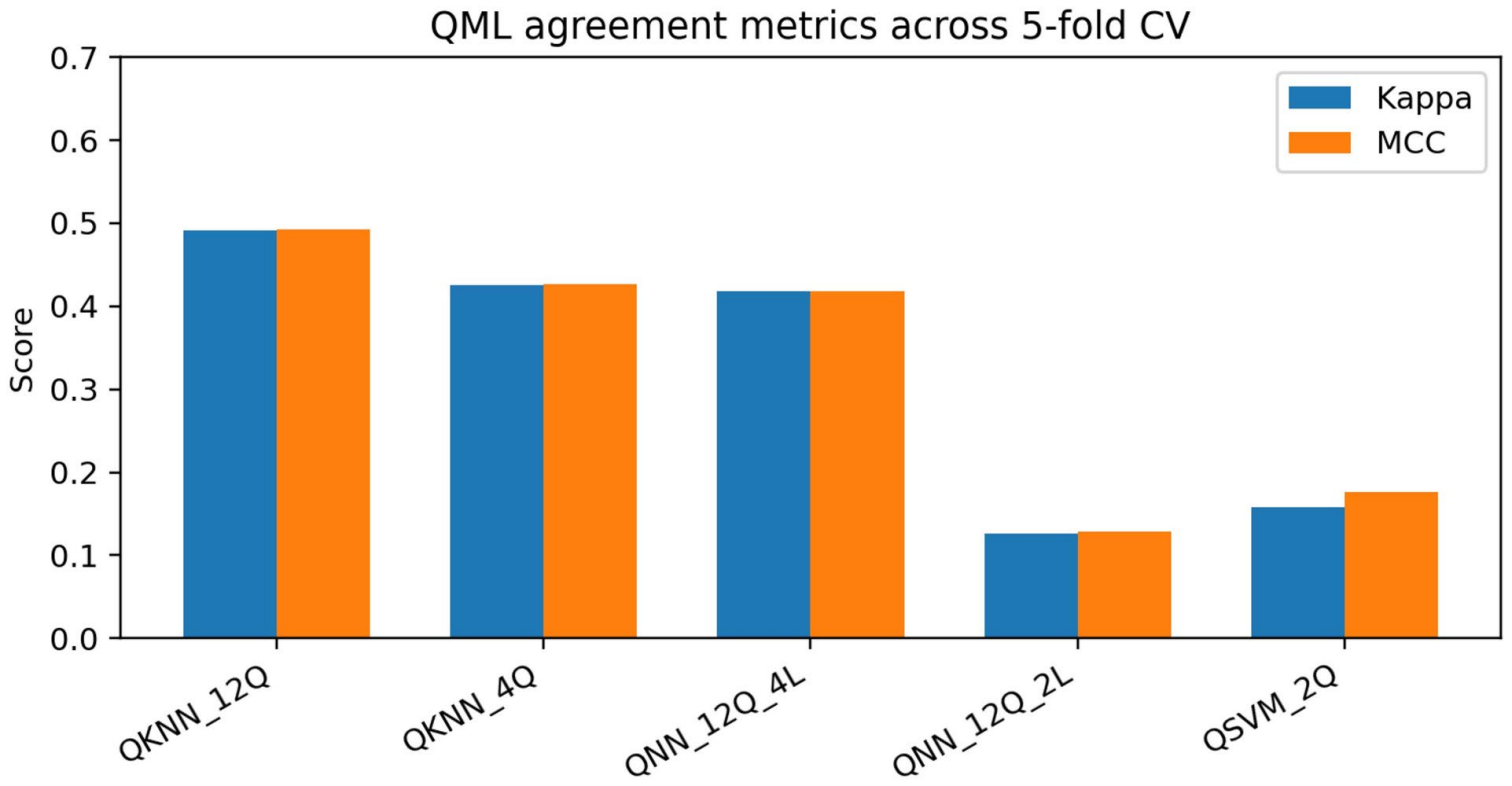
Statistical agreement for QML models, reporting mean Cohen’s Kappa and Matthews correlation coefficient across cross-validation folds.

Youden’s Index, it also nudges the interpretation toward carefulness. QKNN 12Q reached 0.4895 ± 0.0753, QKNN 4Q reached 0.4236 ± 0.0410, and QNN 12Q 4 L reached 0.4183 ± 0.0450. Taken together, these outcomes point to non random discrimination, yet they still do not really support claims that are close to clinical level strength (see Figure 4).

## 6 Discussions

### 6.1 Interpretation of results and practical implications

The results consistently demonstrate that simulator-based QML models remain competitive for exploratory benchmarking but are clearly outperformed by strong classical models trained on the complete feature representation. Rather than indicating quantum advantage, the findings identify the principal limitations that currently constrain practical QML applications for clinical tabular data.

Previously, the linear projection from PCA would have been used to convert QKNN performance into what would be considered “full feature” clinical performance. This over-extrapolation has been eliminated. But not only is it not justified, the relationship between retained variance and prediction quality is not linear, and varies by model family as well. That is, it is only justified on the basis of what we have seen in the cross validation.

In the QML set, the QKNN setups outperformed the QNN and QSVM setups with respect to the accuracy in addition to agreement style metrics. In the meantime, the probability calibration of QNN 12Q 4 L was improved over QKNN. This points to the idea that distance based quantum approaches and variational circuits are good at slightly different things, but neither one, at the moment, surpasses the expanded classical baselines.

QKNN consistently achieved the strongest performance among the evaluated QML models because quantum fidelity preserves local relationships between samples more effectively after dimensionality reduction. Since PCA substantially compresses the original feature space, distance-based classification remains comparatively robust under reduced-dimensional representations. In contrast, the QNN requires optimization of variational quantum circuits, which is susceptible to optimization difficulties such as barren plateaus and sensitivity to parameter initialization. The QSVM is additionally constrained by the use of only two principal components, resulting in substantial information loss before kernel construction. These observations suggest that, under the current simulator-based setting and limited qubit resources, quantum distance-based learning is more robust than variational or kernel-based quantum classifiers.

This study aims to therefore create a clear reference to the simulator based QML behavior on an actual medical dataset. The outcomes highlight that dimensionality reduction, probability calibration, simulator overhead, and the design of the validation are important issues that need to be addressed prior to making more forceful conclusions.

### 6.2 Limitations and current challenges

Several limitations should be acknowledged, and by all means first, the timing results were from experiments conducted within a classical simulation/analytical kernel environment, which is not really related to the execution of physical quantum hardware. Second, the variance in the features is considerably reduced by the PCA dimensionality reduction and this, in turn, has a significant impact on the behavior of the models. The full feature classical baselines are really outperforming the QML models by a lot. In greater detail, there are approximately 44 to 84 percent variance lost depending on the number of qubits in the system after PCA dimensionality reduction. The complete feature classical baseline results clearly illustrate that this loss is not just a theoretical problem – it is also a practical problem that reduces classification accuracy. There are several directions for future work, such as features selection techniques, problem specific encodings, and representations that are more hardware compatible and contain more discriminatory information.

The quantum models exhibit training instabilities as well, including sensitivity to initialization, difficulty to obtain a solution to the optimization problem, and, possibly, barren plateau effects in deeper circuits. The QGAN discriminator is particularly sensitive to the small number of epochs in the adversarial training budget of 5, implying that it is treated as an exploratory negative outcome. Lastly, QML is missing a proper hyperparameter tuning or even architecture guidelines for medical table datasets. Things such as circuit depth, feature maps, entanglement patterns, and the optimizer used, have remained largely empirical. This makes generalisation to the outside of the tested METABRIC setting extremely difficult. Another restriction is we do not have physical quantum hardware validation. The current results are entirely based on simulators and analytical kernels, noise, queue latency, shot count, connectivity of the devices, and any error mitigating effects are beyond the scope of this study and will be looked at later.

In practice, further challenges arise on today’s quantum hardware beyond those captured in simulation. Current NISQ devices are affected by gate errors, qubit decoherence, limited qubit connectivity, finite shot counts, calibration drift, and queue delays; these can reduce predictive performance and increase execution variability. These problems can be reduced to some extent by error mitigation techniques, but hardware noise is not completely eliminated. Hence, the results obtained from experiments in the simulator should be considered as an upper-bound estimate of the algorithmic behavior, and not as an indication of the expected performance on real devices.

### 6.3 Future directions and research opportunities

Future studies should focus on hybrid quantum classical methods, that combine quantum feature extraction or quantum kernels with strong classical decision functions. Designs like that may allow QML parts to be tested, without having to replace well established classical healthcare ML pipelines, entirely, kind of.

Problem specific quantum feature maps should also be explored. Instead of trusting only generic angle embedding, future work can look at encoding strategies that keep clinically meaningful feature interactions intact, and also reduce the information loss that comes from PCA, which is a big deal.

Testing on real devices should still happen. Future work should specify the hardware backend, the noise model, the number of shots, the queue-independent runtime, the error mitigation technique, and comparison with simulations before concluding about deployment. The learning curves and sample efficiency studies comparing QML and classical models over dataset sizes can clarify if QML has a benefit for a specific data size and feature geometry, without making “quantum advantage” claims *a priori*.

Interpretability and explainability methods development are required in order to secure the clinical acceptability and comply with the regulatory standards for medical artificial intelligence. Besides the performance level, quantum models treated as black boxes have huge resistance to being clinically adopted. These decision rules extraction techniques, quantum feature spaces visualizing, and prediction uncertainty quantifying must be developed along with performance gains.

There lies a necessity of research for optimization approaches that will include the quantum natural gradients, the meta-learning for quantum circuits, the neural architecture search for quantum circuit design, and the better initialization strategies. The extension to multi-class and multi-label issues would allow the handling of different breast cancer sub-types, simultaneous multiple predictions, survival analysis integration, and uncertainty quantification to be incorporated thus wide-spreading the applicability.

In the healthcare quantum computing community, people really need stricter evaluation standards, like more solid classical baselines, plus cross validation and some proper statistical testing. Future research should adopt more rigorous evaluation protocols, including stronger classical baselines, stratified cross-validation, statistical significance testing, calibration analysis, transparent reporting of feature compression, and hardware-aware runtime interpretation. Such practices are essential before drawing conclusions regarding potential quantum advantage in healthcare applications.

## 7 Conclusion

This study looked at five QML configurations for METABRIC breast cancer classification. It did this under strict rules using PCA and a simulator. At the time it also used seven other classical methods for comparison. The way the study was set up was a 5 fold cross validation. This means that the preprocessing was done for each part separately and the classical comparisons were matched to PCA. There were also comparisons using all 63 features for contrast. For the QML models QKNN 12Q gave the results overall. It got 75.11% accuracy, an F1-score of 0.7088 and ROC-AUC of 0.8148. QKNN 4Q and QNN 12Q 4 L also showed some promise. Qnn 12Q 2 L and QSVM 2Q were not as strong. The main take away is that the classical methods using all the features did better than the QML models. XGBoost Random Forest and Logistic Regression got around 94% accuracy when using all 63 features. So this study does not really show that QML models are better and it should not be seen as a claim that they are ready for use in clinics.

The results further demonstrate that information loss caused by PCA is a major limitation of current simulator-based QML workflows. While classical models achieved approximately 94% accuracy using the complete 63-feature representation, their performance declined to around 82% after PCA-12 compression, and the best-performing QML model (QKNN 12Q) achieved 75.11% accuracy. These findings indicate that dimensionality reduction, rather than the quantum models alone, is a primary factor limiting predictive performance. Likewise, the reported inference times represent simulator or analytical-kernel execution and should not be interpreted as evidence of quantum computational advantage. Validation on real quantum hardware, together with noise characterization and error-mitigation strategies, remains essential before drawing conclusions regarding practical deployment or scalability.

Overall, this work provides a controlled and reproducible benchmark for evaluating current quantum machine learning architectures on a real-world breast cancer dataset under realistic computational constraints. Rather than claiming quantum advantage or clinical readiness, the study identifies the key factors that currently limit QML performance, including feature compression, restricted qubit resources, simulator overhead, and optimization challenges. Future research should focus on hybrid quantum-classical approaches, hardware-aware validation, improved feature encoding and selection techniques, explainable QML models, and evaluations on real quantum devices. These directions are expected to provide a more realistic path toward assessing the potential role of quantum machine learning in healthcare.

## Data Availability

The original contributions presented in the study are included in the article/supplementary material, further inquiries can be directed to the corresponding author.
